# Trends in the two-component system’s role in the synthesis of antibiotics by *Streptomyces*

**DOI:** 10.1007/s00253-023-12623-z

**Published:** 2023-06-21

**Authors:** Rodrigo Cruz-Bautista, Beatriz Ruíz-Villafán, Alba Romero-Rodríguez, Romina Rodríguez-Sanoja, Sergio Sánchez

**Affiliations:** Instituto de Investigaciones Biomédicas, Universidad Nacional Autónoma de México, Ciudad Universitaria, CdMx, 04510 Mexico City, Mexico

**Keywords:** *Streptomyces*, Two-component systems, Antibiotics, Regulation, Sensor histidine kinase, Response regulator

## Abstract

**Abstract:**

Despite the advances in understanding the regulatory networks for secondary metabolite production in *Streptomyces*, the participation of the two-component systems (TCS) in this process still requires better characterization. These sensing systems and their responses to environmental stimuli have been described by evaluating mutant strains with techniques that allow in-depth regulatory responses. However, defining the stimulus that triggers their activation is still a task. The transmembrane nature of the sensor kinases and the high content of GC in the streptomycetes represent significant challenges in their study. In some examples, adding elements to the assay medium has determined the respective ligand. However, a complete TCS description and characterization requires specific amounts of the involved proteins that are most difficult to obtain. The availability of enough sensor histidine kinase concentrations could facilitate the identification of the ligand–protein interaction, and besides would allow the establishment of its phosphorylation mechanisms and determine their tridimensional structure. Similarly, the advances in the development of bioinformatics tools and novel experimental techniques also promise to accelerate the TCSs description and provide knowledge on their participation in the regulation processes of secondary metabolite formation. This review aims to summarize the recent advances in the study of TCSs involved in antibiotic biosynthesis and to discuss alternatives to continue their characterization.

**Key points:**

*• TCSs are the environmental signal transducers more abundant in nature.*

*• The Streptomyces have some of the highest number of TCSs found in bacteria.*

*• The study of signal transduction between SHKs and RRs domains is a big challenge.*

**Supplementary Information:**

The online version contains supplementary material available at 10.1007/s00253-023-12623-z.

## Introduction

The *Actinobacteria* family includes the genus *Streptomyces*, Gram-positive bacteria with high GC DNA content, a linear chromosome ranging from 6.7 to 12.3 Mb, and a complex life cycle. *Streptomyces* can be found in soil, sea, and riverbeds (Chater 2016; Sivalingam et al. 2019). They are known for producing over two-thirds of the antibiotics used in the clinic (Yin et al. 2019). Environmental and physiological stimuli are critical in morphological and chemical (antibiotic production) differentiation (Hoskisson and Fernández-Martínez 2018). These stimuli must be correctly sensed to elicit an adequate response. This answer is achieved by transcriptional factors (TFs) through signal transduction pathways using one or two-component systems (TCSs) (Romero-Rodríguez et al. 2015). The latter are typically composed of two proteins. One is a transmembrane sensor histidine kinase (SHK), which transfers a phosphate group from a histidine moiety to an aspartate residue on the second protein. This last protein is a cognate response regulator (RR) that can exert an effect at the level of its target genes (Jung et al. 2018).

TCSs are widespread among microorganisms perceiving environmental signals to trigger cellular responses that allow them to face ecological changes. These signals can be of chemical or physical nature, and by sensing these stimuli, bacteria can acquire nutrients, respond to stress, and exchange information with other cells to adapt and survive within their ecological niche or in case of pathogens and commensals within their host (Jacob-Dubuisson et al. 2018; Jung et al. 2018). Intracellular pathogen bacteria living in a homeostatic environment encode only a few TCSs. In contrast, in bacteria living in a competitive one, like *Streptomyces*, TCSs are abundant and essential to adapt to environmental and nutritional changes rapidly.

Advances in the TCSs research in several streptomycetes (e.g., *Streptomyces coelicolor*, *Streptomyces avermitilis*, *Streptomyces antibioticus*, and *Streptomyces lividans*) permitted the use of metabolic engineering to increase their efficiency in the production of antibiotics, as well as to discover cryptic antimicrobials not produced under laboratory conditions (Rodríguez et al. 2013). Of the reported TCSs in *Streptomyces*, only a few are known to be characterized by the events which start with the induced signal to the triggered regulatory response (McLean et al. 2019a; Sánchez de la Nieta et al. 2022; Jin et al. 2023). In general, the characterization of a TCS consists of studying four main areas: signal detection by the SHK, kinase activation, phospho-transferring to its cognate RR, and response generation. Each of these steps brings different challenges. One additional challenge lies in its nature due to the SHK complex structure with extra cytoplasmatic, transmembrane, and cytoplasmatic domains (Zschiedrich et al. 2016).

On the other hand, studying RRs becomes complicated when their conformationally dynamic nature is studied. Still, in the case of the regulatory domains (REC), which are conserved but dynamic, the use of many RR structures allowed the description of their interaction with DNA, SHKs, and auxiliary proteins (Gao et al. 2019). Recently, various techniques have been developed and used for TCS studies. These included phosphorylation monitoring, design and purification of tagged fusion proteins, site-directed mutagenesis, ligand binding assays, and, more recently, bioinformatics tools, among others (Scharf 2010; Perry et al. 2011; Hussain et al. 2016). The progress of the study of TCS in regulating antibiotic production in the genus *Streptomyces* is summarized in Table S1.

This review aims to summarize the TCSs involved in antibiotic production in *Streptomyces* and discuss the main challenges and most recent methodologies for TCSs characterization in these bacteria.

## General characteristics of the two-component systems

TCSs are the environmental signal transducers more abundant in nature, enabling bacteria to adapt to various environmental changes. This system is ubiquitous in bacteria, including commensal, pathogen, or free-living organisms, but it is not present in mammals (Padilla-Vaca et al. 2016). However, some eukaryotic cells, such as fungi, yeast, and several higher plant cells, possess similar systems (Zhao et al. 2022).

**Fig. 1:**
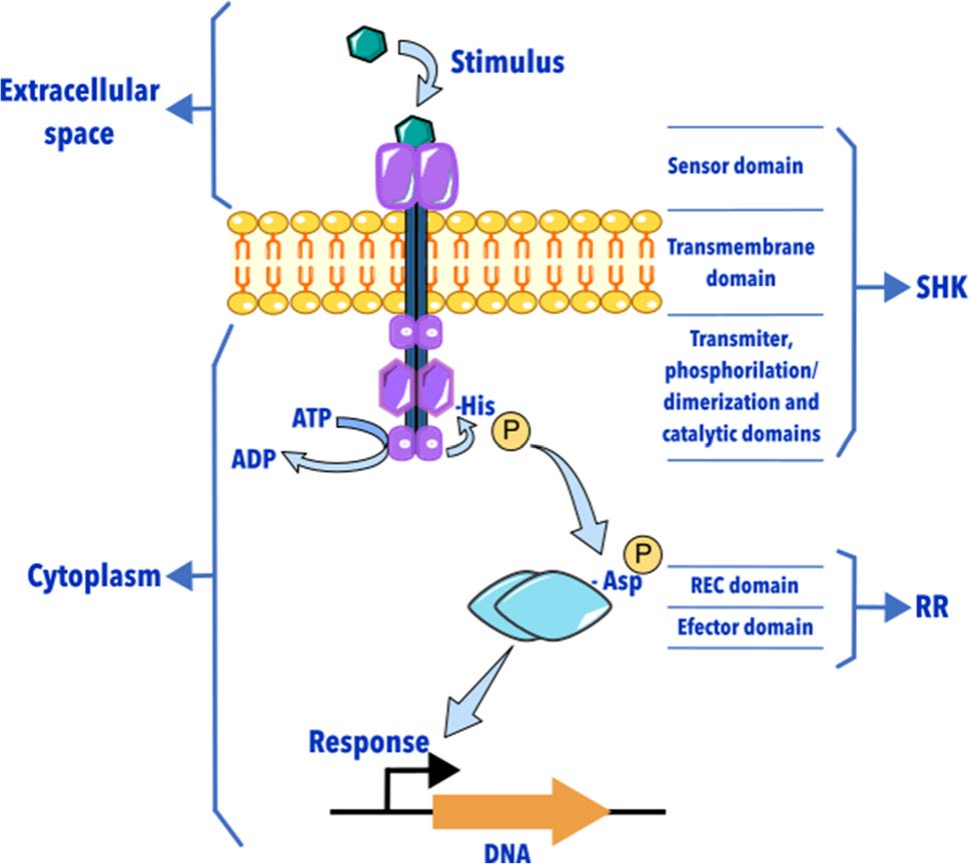
Schematic representation of a classic two-component system. SHK senses the stimulus, transferring it through the membrane to the catalytic domain. Here a phosphate group from ATP is moved to a conserved histidine residue. Then, the phosphate group is passed to an aspartate residue in the RR, eliciting the regulatory response to the stimulus

TCSs, in their most common and straightforward organization, comprise a sensor histidine kinase (SHK) and its cognate cytoplasmatic response regulator (RR). They are present as operonic pairs in bacterial genomes, but orphan (unknown pair) SHKs and RRs are also found (Padilla-Vaca et al. 2016). TCSs evolved to sense many stimuli such as light, temperature, pH, metals, nutrient availability, respiratory electron acceptors, oxidizing agents, small-molecule metabolites, inter-bacterial communication signals, antibiotics, antimicrobial peptides, oligosaccharides, proteins, hormones, and other host-derived signals (Lazar and Tabor 2021a, b). The TCSs rely on phosphoryl-transfer reactions to transmit information. The stimulus–response mechanism begins when the SHK senses a stimulus and transmits the signal to a RR, which commonly binds to DNA to control the cellular response (Jacob-Dubuisson et al. 2018) (Fig. 1). For a given signal, the TCSs evolved to elicit efficient, specific, and adequate directionality responses.

### Sensor histidine kinases

The SHKs are transmembrane proteins with an extracellular sensor domain. They require the formation of homodimers to transmit the signal across the membrane. SHKs transfer a phosphoryl group from ATP to one of its conserved histidine residues by a *cis* or *trans* mechanism (each monomer phosphorylates itself or the other) (Padilla-Vaca et al. 2016; Zschiedrich et al. 2016). Then, this amino acid moves this group to an aspartate residue in the RR (some TCSs have serine/threonine phosphorylation). To regulate this response, many SHKs also have a phosphatase function to cleavage the phosphoryl group from the aspartate residue in the RR. SHKs are multi-domain proteins with sensor, transmembrane, signal transducing, dimerization, histidine phosphotransferase, and catalytic domains. Usually, SHKs have extracellular sensor domains, but these can also be transmembrane or intracellular (Bhate et al. 2015).

Some SHKs are highly specific for a single input, while others sense multiple inputs characteristic of a particular environment. Sensing of numerous inputs require multiple binding sites in single or multiple sensor domains (Lazar and Tabor 2021a, b).

### Response regulators

RRs usually comprise two domains, a conserved N-terminal receiver domain (REC) and a more variable C-terminal effector domain (Fig. 1). REC catalyzes the transfer of the phosphoryl group from SHK. Homo-multimer or dimer-formation depends on the variable C-terminal effector domain, and its activation works in a phosphorylation-dependent manner (Zschiedrich et al. 2016). Phosphorylation occurs canonically in a highly conserved aspartate residue. It allows the formation of a stable and active structure, enabling the effector domain function (Gao et al. 2019). We know that 70% of RRs bind to DNA to exert their function, but others act through binding to RNA, proteins, or by enzymatic activity (Zschiedrich et al. 2016).

## TCSs in *Streptomyces*

The *Streptomyces* bacteria have some of the largest genomes and the highest number of TCSs found in bacteria, perhaps reflecting an adaptation to their highly variable niche (Romero-Rodríguez et al. 2015). Indeed, these microorganisms have inhabited challenging environments, forcing them to develop complex responses that allow them to adapt, making TCSs an essential part of this process. On average, the *Streptomyces* genus, estimated by genome sequence analysis, has 90 sensor kinases and 80 regulatory proteins, including unpaired SHKs and orphan RRs (Romero-Rodríguez et al. 2015). A more recent bioinformatic analysis using the online tool P2RP in 93 complete genomes of different *Streptomyces* species predicts 69 TCSs, 109 SHKs, and 87 RRs in *S. coelicolor*, being 21 orphan RRs and 39 unpaired SHKs (McLean et al. 2019a). The abundance of TCSs makes evident the importance of these signal transduction systems for *Streptomyces* (Rodríguez et al. 2013).

## Regulation of antibiotic biosynthesis by TCSs in response to different stimuli

As we have mentioned before, the availability of carbon, phosphate, or nitrogen sources in the media can affect the production of secondary metabolites. Variations of these and other environmental elements, like metallic ions, antibiotics, and butyrolactones, are sensed by *Streptomyces*, modifying the production of antibiotics (Romero-Rodríguez et al. 2018). The TCSs studied so far are divided according to the stimulus that may trigger their activation and response. These included those where the apparent elicitor is not defined even when being studied.

### Phosphate

Phosphate is an essential nutrient for most life forms and is necessary for synthesizing cell membranes, genetic material, energy carriers, metabolism, and metabolic signaling (Park et al. 2022). It is well established that phosphate starvation triggers changes in the metabolism of antibiotic-producing microorganisms, causing the slowdown of primary metabolism and increasing secondary metabolites production (Martín et al. 2017). Consequently, the industrial production of secondary metabolites requires low amounts of phosphorus in the fermentation tanks, although biomass production can be reduced (Romero-Rodríguez et al. 2018).

**Fig. 2:**
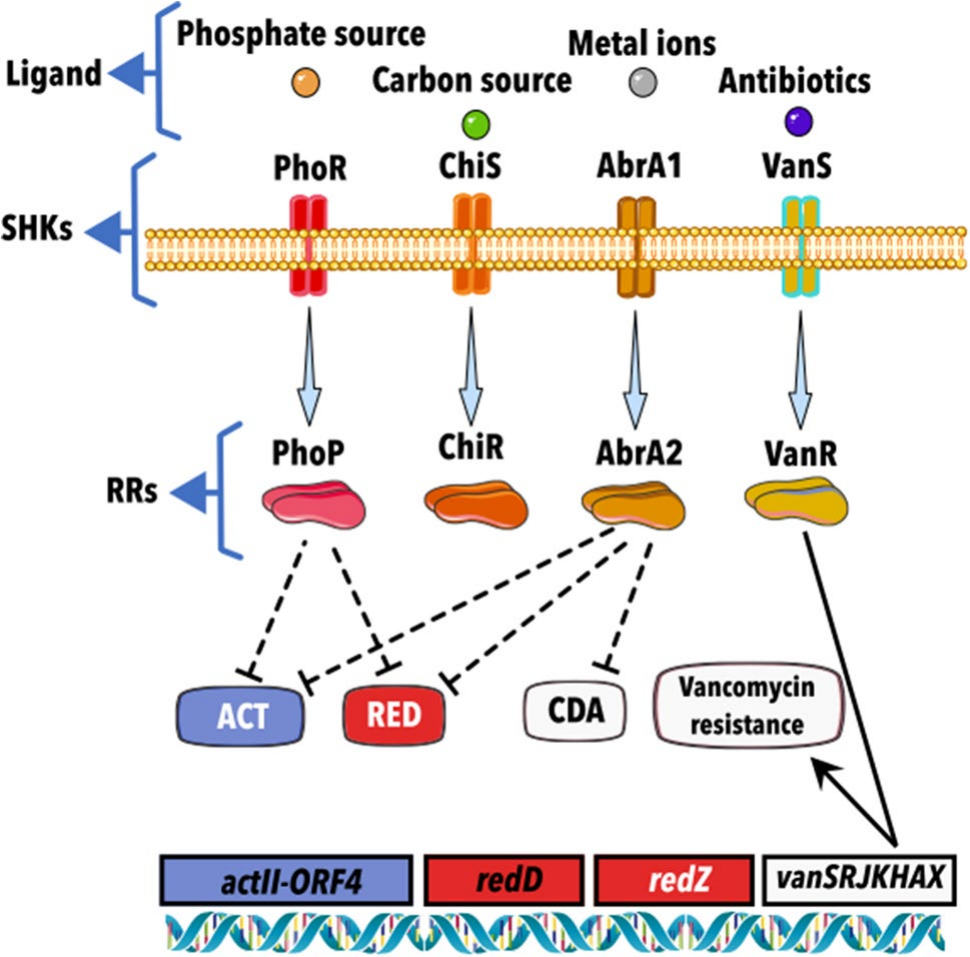
Schematic representation of the regulation exerted by two-component systems on different systems using different trigger signals. An arrow connects the Sensor Histidine Kinases and their cognate response regulators. Lines ending in an arrow indicate a positive regulation, and truncated lines indicate a negative regulation. Continuous lines indicate a direct effect through genes involved in synthesizing or regulating the metabolite, and discontinuous lines indicate indirect or unknown regulation. ACT, actinorhodin; RED, undecylprodigiosin; and CDA, calcium-dependent antibiotic

The Pho regulon responds to inorganic phosphate starvation, being regulated by a TCS called PhoR-PhoP (Fig. 2) (SHK/RR) (Santos-Beneit 2015). PhoR phosphorylates PhoP to activate the *pho* regulon (Fernández-Martínez et al. 2012). Experimental evidence in *S. coelicolor*, using reverse transcription-polymerase chain reaction (RT-PCR) and reporter luciferase, suggested that PhoR can also phosphorylate itself. On the other hand, the heterologous expression of PhoP and its truncated DNA binding domain for footprinting assays showed that PhoP could recognize and bind to PHO boxes, which are well-conserved repeated units of 11 nucleotides present in a great variety of genes (Sola-Landa et al. 2005). These genes involve phosphate scavenging, transport, storage, mobilization, oxidative phosphorylation, nitrate respiration, protein synthesis, and RNA polymerases (Martín et al. 2017). Under phosphate starvation, PhoP also regulates undecylprodigiosin (RED) and actinorhodin (ACT) (Martín et al. 2017). Binding assays have shown that regulation of antibiotic formation occurs on AfsS (which activates the expression of *actII-orf4* and *redD*) by a competitive effect between AfsR (pleiotropic regulator phosphorylated by a serine/threonine kinase AfsK) and PhoP. Besides, the assays also indicated that PhoR-PhoP is regulated by AfsR, establishing in this way a cross-interaction (Santos-Beneit et al. 2009).

Commercial and prototypical compounds useful in the clinic are strongly influenced by phosphate starvation. In this aspect, several TCSs have also been identified. For example, in *Streptomyces roseosporus*, the AtrA regulator (involved in the production of daptomycin) is regulated by PhoP by partially overlapping between their binding sites located at the *atrA* promoter (Zheng et al. 2019).

In *Streptomyces hygroscopicus* var. *geldanus*, PhoP has an early effect (7.5 h post-starvation) in the type III polyketide synthetase gene (*sco7221*) involved in the synthesis of germicidin and in the *cpk* genes involved in the synthesis of coelimycin P1 (Martín et al. 2017). Also, PhoP plays a key role in the biosynthesis of geldanamycin, whose regulation is quite sensitive to the inorganic phosphate concentration. Deletion of PhoP in this strain prevented their growth in soya peptone, glucose, and zinc sulfate medium (Martín et al. 2019). In the tacrolimus producer *Streptomyces tsukubaensis*, STSU_19405 and STSU_19410 have been identified as the putative orthologs for PhoR/PhoP. A bioinformatic search of PHO boxes performed in *S. coelicolor* and complementation studies demonstrated the functionality of STSU_19410 (Ordóñez-Robles et al. 2017).

### Carbon source

The presence of rapidly metabolized carbon sources like glucose promotes rapid growth and simultaneously represses the catabolism of other carbon sources. This process is regulated by carbon catabolite repression (CCR) (Simpson-Lavy and Kupiec 2019). For *Streptomyces*, it has been proved that different carbon sources inhibit the synthesis of secondary metabolites (Romero-Rodríguez et al. 2018). However, a correlation between this effect and the CCR mechanism has not been fully established (Ruiz-Villafán et al. 2021). Only a few examples of TCSs’ participation in *Streptomyces* responding to the presence of carbon sources in the medium have been identified, and most are not well characterized. In the natural environment, chitin and N-acetylglucosamine from crustaceans, fungi, and insects are the most abundant nutrient sources for *Streptomyces* (McLean et al. 2019a). Secreted chitinase is the most abundant enzyme in streptomycetes. In *Streptomyces thermoviolaceus* OPC-520, the TCS ChiS/ChiR (SHK/RR) (Fig. 2), coded upstream of *chi40*, enhances enzyme production. Besides, enzyme production is induced by chitin and chitobiose in *S. lividans* transformed with *ChiS/ChiR/chi40* genes (Tsujibo et al. 1999). Furthermore, the induction of the chitinase gene *chiC* in *S. coelicolor* by chitin and its reduction in a *chiR-*disrupted strain were demonstrated using *XylE* as a reporter. However, it was impossible to show that *chiR* can bind to the promoter region of *chiC* (Homerová et al. 2002). An interaction between the SHK of these TCSs and the chitobiose-binding protein DasA has been suggested in the chitinolytic system of *Vibrio* species (Colson et al. 2008) but, as far as we know, it has not been further characterized. In *Streptomyces peucetius*, the autophosphorylation of ChiS was determined to be in the 144-histidine residue (Rabbind-Singh et al. 2014). Although there is no evidence of direct regulation of antibiotic production by this TCS, chitin in the medium is known to induce ACT and RED production in *S*. *coelicolor* (Nazari et al. 2013).

Under laboratory conditions, the preferred carbon source for *Streptomyces* growth is glucose. High concentrations of this sugar interfere with the biosynthesis of secondary metabolites and morphological differentiation (Ruiz-Villafán et al. 2021). The effect of glucose has been studied by a transcriptomic analysis performed in the wild-type M-145 strain of *S. coelicolor* and a *glk* null-mutant (Δ*glkA*) derivative complemented with the *glk* gene from *Zymomonas mobilis* grown under repressive (glucose 0.5% + agarose 0.5%) and non-repressive conditions (agarose 0.5%) (Romero-Rodríguez et al. 2016b). From this analysis, the following four TCSs appear to be influenced by the presence of this carbon source in the media. (i) *sco5784*/*sco5785* (SHK/RR) is a TCS involved in the positive modulation of ACT biosynthesis and the regulation of the transition from the primary to secondary metabolism in *S. coelicolor* (Rozas et al. 2012). (ii) *sco6162/sco6163* (RR/SHK) was found to be involved in the negative effect exerted by glucose in the biosynthesis of ACT and RED. This effect is now under characterization in our research group (Cruz-Bautista et al. in preparation). (iii) *sco3134* encodes a putative orphan RR belonging to the LuxR family, and* (iv) sco4020*/*sco4021* (RR/SHK), a non-characterized TCS (Romero-Rodríguez et al. 2016a).

Thus, it would be worth continuing to characterize it to establish a possible correlation between them and the carbon source present in the medium.

### Nitrogen source

Glutamate and glutamine are essential amino acids for transamination reactions to form different nitrogen-containing compounds in *Streptomyces* and other microorganisms. These amino acids are obtained from various single nitrogen sources, such as ammonium ions, nitrate, and different complex sources, like urea, and peptones (Martín and Liras 2020). Although some TCSs have been identified as nitrogen ligands, sometimes it appears that the relationship between N/P/C is the one that genuinely regulates these systems (Rodríguez et al. 2013). Those TCSs seem to be influenced by the presence of nitrogen sources are mentioned below.

**Fig. 3:**
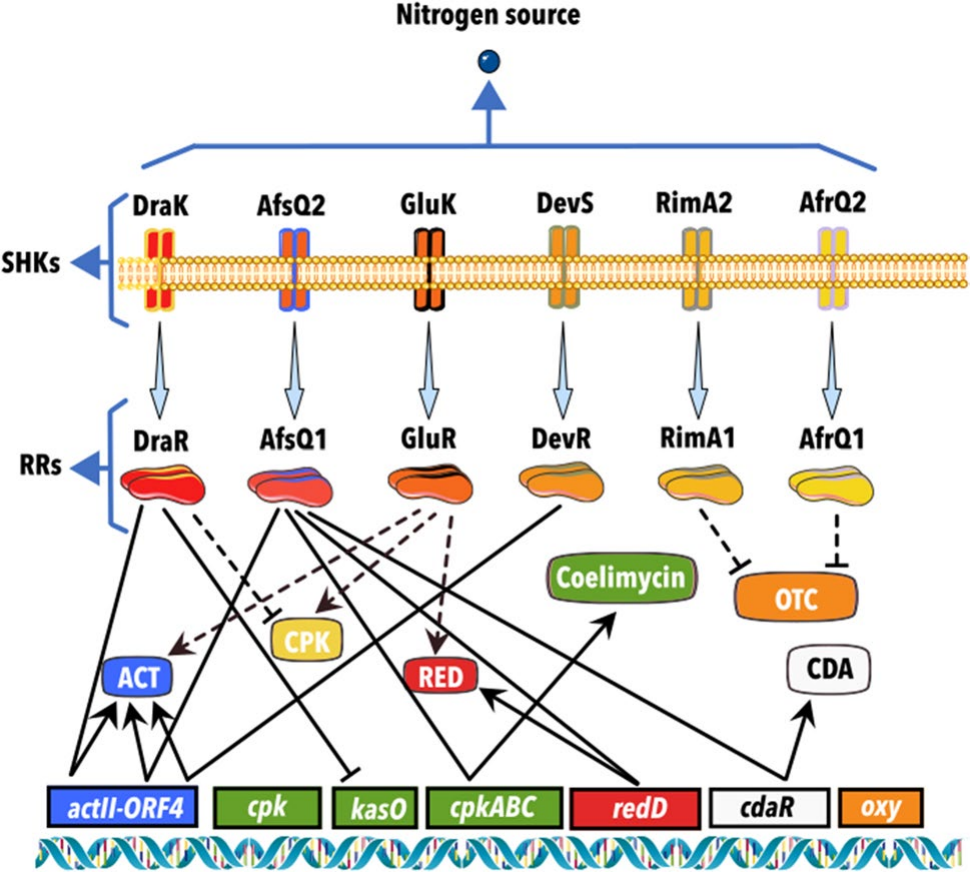
Schematic representation of regulation pathways by the two-component systems whose trigger signal has been identified as a nitrogen source. An arrow connects Sensor Histidine Kinases and their cognate response regulators. Lines that end with an arrow indicate a positive regulation, and truncated lines indicate a negative regulation. Straight lines indicate a direct effect through genes involved in synthesizing or regulating the metabolite, and dotted lines indicate indirect or unknown regulation. ACT, actinorhodin; RED, undecylprodigiosin; CPK, coelimycin polyketide synthase I; and CDA, calcium dependent

Dra-R/K (RR/SHK) is a TCS encoded by *sco3063*/*sco3062* in *S*. *coelicolor* (Fig. 3) involved in the positive regulation of ACT and the negative regulation of RED (Sánchez de la Nieta et al. 2020). Likewise, when high nitrogen concentrations are present in the medium (amino acids), it activates ACT and represses yCPK (yellow-pigmented type I polyketide) production through the *actII-ORF4* and *kasO* genes, respectively. On the contrary, RED is repressed by an independent *redD/redZ* pathway (Yu et al. 2012). Also, the identification of the consensus DNA-binding site for DraR by DNase I footprinting assays and a genome screening performed by gel-retardation and functional analysis allowed the detection of the target genes involved in primary metabolisms such as glutamate synthase *gltB* (*sco2026*), pyruvate kinase (*sco2014*), and a probable aminotransferase (*sco6222*), among others (Yu et al. 2012). In the same study, it was found that DraR is negatively auto regulated. In the case of the *S*. *avermitilis DraR* homolog, this negatively regulates avermectin production and positively the synthesis of oligomycin A. An interesting interaction between DraR-K and AfsQ1-Q2 was observed in a double mutant (*ΔdraR*-*afsQ1*) by comparing its *actII-ORF4* transcriptional levels with those of a *ΔdraR* mutant, suggesting that AfsQ1 may be an activator that cooperates with DraR in the regulation of ACT in a transcriptional level (Yu et al. 2012). Later, the transcriptomes of the *S*. *coelicolor* M145 and the *ΔdraR-K* strains, grown in minimal medium (MM) supplemented with glutamine, showed that genes involved in geosmin, carotenoids, siderophores, and tetrahydroxy-naphthalene biosynthesis were also affected (Yu et al. 2014). Additionally, in search of understanding the activation conditions of this TCS, it was performed a structural study of the sensory domain of SHK DraK using nuclear magnetic resonance (NMR) and circular dichroism (CD) spectroscopy. This study suggested that the structure of its extracellular sensor domain is pH-dependent, being more structured at low pH and that the glutamate at position 83 is essential for this conformational change (Yeo et al. 2013).

The deletion of the TCS AfsQ1-Q2 (RR-SHK) in *S. coelicolor* (Fig. 3) grown in MM supplemented with glutamate showed that this TCS positively regulates the expression of ACT, RED, CDA (directly by *actII-ORF4*, *redD*, and *cdaR*), and coelimycin P2 (Shu et al. 2009; Chen et al. 2016). Interestingly when the sigma factor *sigQ* (located upstream *afsQ1-afsQ2*) was also deleted, there was an early overproduction of these antibiotics. RT-PCR analysis and enhanced green fluorescent protein (EGFP) fusion assays suggested that *sigQ* is under the control of *afsQ* (Shu et al. 2009). Moreover, by footprinting, electrophoretic mobility shift assays (EMSA), and site-directed mutagenesis, the binding sites for these genes were determined (Wang et al. 2013). The homolog of this TCS in *Streptomyces albus* is RspA1/A2, which plays a regulatory role in salinomycin biosynthesis in this species. Deletion of RspA1/RspA2 supported that this TCS promotes salinomycin production by the specific activator gene *slnR* (Zhang et al. 2021). RT real-time PCR (RT-qPCR) and EMSA experiments also showed that this RR directly activates *sigW* (Sigma factor located downstream of rspA1). This sigma factor transcriptionally represses nitrogen assimilation by interacting with the promoter regions *glnA*, *amtB*, *gdhA*, *SLNWT_1828* (urea carboxylase), and *SLNWT_1829* (allophanate hydrolase) (Zhang et al. 2020). It is also worth mentioning that this TCS has an impact on the regulation of glucose metabolism (Zhang et al. 2020).

In a bioinformatic analysis, the GluR-GluK (*sco5778*-*sco5779*) was identified as a TCS in *S*. *coelicolor* (Fig. 3). This system appears to be widely distributed in Actinobacteria (Li et al. 2017b). GluR is an OmpR RR, and GluK is a typical transmembrane SHK. Experiments on MM supplemented with glutamate showed that this TCS positively affects RED and yCPK biosynthesis but negatively affects ACT production (Li et al. 2020). GluR regulates the *gluABCD* operon (which is located divergently from this TCS) by binding in a motif upstream of *gluA*. The observation that GluR cannot bind to the classical regulators of antibiotics (*redZ*/*redD*, *kasO*, and *actII-ORF4*) in EMSA experiments suggests that this regulation is mediated indirectly (Li et al. 2017b).

In *Streptomyces rimosus* M4018, the TCS RimA1/A2 (Fig. 3) was identified to have homology with the TCS RapA1A2 in *S*. *coelicolor* (82% and 71%, respectively). As shown in the same figure, *rimA1* encodes for a RR of the OmpR family, and *rimA2* for a protein with the typical properties of an SHK, containing the H box and motifs N, G. A mutant strain in *rimA1* tested on solid and in liquid MM supplemented with glycine as the only nitrogen source exhibited an increase in the production of oxytetracycline (OTC). RT-PCR assays demonstrated overexpression of the oxy gene cluster (*oxyB*, *otrB*, *otcG*, and *otcR*). Overexpression of RimA1/A2 negatively regulates OTC production in MM with glycine in *S. rimosus*. On the contrary, when grown under stress conditions in the presence of KCl (high osmolarity), an increase in the OTC yield was observed. These findings suggest a negative regulation of the production of OTC and a positive role in osmotic stress adaptation (Ni et al. 2019). Another TCS involved in OTC biosynthesis in this strain is AfrQ1Q2 (Fig. 3). This TCS, also identified by bioinformatic analysis, is a homolog to AfsQ1Q2 of *S*. *coelicolor*. It was shown by RT-PCR that both *afrQ1* (RR) and *afrQ2* (SHK) are co-transcribed and that disruption of *afrQ1* resulted in the enhancement of OTC production in MM with glycine as the sole nitrogen source. RT-qPCR assessed the transcriptional levels of five genes related to the biosynthesis and regulation of OTC (*oxyB*, *otrB*, *otcG*, *otcR*, and *otrC*). The results supported a clear up-regulatory pattern in this mutant compared with the wild type and the complemented strains, suggesting a negative global regulation of OTC (Ni et al. 2020).

Studies of mutants performed by complementation, EMSA, chromatin immunoprecipitation-sequencing (ChIPseq), and qPCR have generated a robust model of DevS/R (Fig. 3) on the regulation of ACT by the nitric oxide (NO) signaling in *S. coelicolor* (Honma et al. 2021). This system comprises a heme-containing NO sensor protein (DevS) and a cognate RR (DevR), which binds directly to *actII-ORF4*. The autophosphorylation activity can be inactivated when NO concentration increases during cell growth. On the other hand, DevR is phosphorylated by DevS in an intracellular NO level-dependent manner, activating its own gene and the *nar2* gene cluster, resulting in a strict autoregulation system for cellular NO homeostasis. Mutants with a significant decrease in their NO-producing ability also showed reduced production of antibiotics and early sporulation (Honma et al. 2021).

### Metal ions

The TCS AbrA1/A2 (*sco1744*/*sco1745*) from *S*. *coelicolor* M145 (Fig. 2) is a negative regulator of ACT, RED, CDA production, and differentiation. The search for the signal that triggers the activation of Abr1 has been made by experiments in which mutant strains were grown in different media supplemented (or not) with phosphate, glucose, (NH_4_)_2_SO_4_, and casamino acids. The observation that both MgSO_4_ and FeSO_4_ are needed for the repression of ACT production in WT strains and that their effects are not additive suggests that one or both elements are the activating signals (Rico et al. 2014a). However, it still needs to be determined if they promote a direct or indirect activation.

AbrA1/A2 is part of an operon with the upstream ABC system encoded by *sco1742* and *sco1743*. A positive autoregulation of this operon was detected using the xylanase reporter gene *xysA*, from *Streptomyces* *halstedii* JM8, under the control of *abrAP* (Rico et al. 2014b). Some crosstalk was proposed in this system considering that there are intermediate phenotypes in the individual mutants (*ΔabrA1* or *ΔabrA2*), which could be due to non-cognate HKs and RRs that may act when the corresponding partners are absent (Rico et al. 2014b).

### Antibiotics

The VanR-VanS (Fig. 2) proteins are encoded by *sco3589* and *sco3590*, respectively. Until now, it is one of the best-characterized TCS in *Streptomyces*. It triggers *vanSRJKHAZ* (four transcriptional units: *vanRS*, *vanJ*, *vanK*, and *vanHAX*) gene transcription, which confers resistance to vancomycin. This cluster is usually present in pathogenic bacteria but is also found in non-pathogenic glycopeptide-producing strains like *S. coelicolor* and *Streptomyces toyocaensis* (Hutchings et al. 2006). VanS acts as a phosphatase in the absence of vancomycin. In the presence of the antibiotic, it works as a kinase, increasing the phosphorylated VanR and activating the transcription of *van* promoters. In *S*. *coelicolor*, in the absence of an inducer, VanR is activated by acetyl phosphate, and VanS acts as phosphatase, suppressing the high levels of phosphorylated RR. Interestingly, in *S*. *toyocaensis*, a difference of eight amino acids in the REC region of the RR allows VanS to be the only one that can phosphorylate (Novotna et al. 2016). A molecular description of the binding site for vancomycin in VanS, located in an extracellular sensor loop between the transmembrane domains (TMD) 1 and 2, was described, revealing an epitope within the N-terminal extracellular region of the protein which adopts an α-helical conformation (Lockey et al. 2020). Also, the X-ray crystal structure of VanR from *S. coelicolor* in active and inactive states has been published, showing the structural transition of helix four situated within the receiver domain. This condition promotes the dimerization of the protein, enhancing the binding ability to the DNA (MacIunas et al. 2021). Moreover, phenotypic and transcriptomic analysis has shown that the TCS AbrB1/B2 (*sco2165*/*sco2166*) is involved in the negative regulation of antibiotic production but positively regulates vancomycin resistance. Deletion of the complete TCS by the CRISPR-Cas9 system in *S*. *coelicolor* M145 grown in liquid and solid NMMP media enhanced the synthesis of ACT and RED by 20- and tenfold, respectively. RNA-seq studies suggested this TCS's contribution to vancomycin resistance (Sánchez de la Nieta et al. 2020). Besides, it was observed that this controlled the basal levels of the vancomycin gene cluster. Since vancomycin itself induces the *S. coelicolor* resistance through the VanRS TCS, the same authors compared the survival rates of the WT strain and the *∆abrB* mutant in the presence of vancomycin. The mutant resulted more susceptible to vancomycin (Sánchez de la Nieta et al. 2020).

### Unidentified stimulus

**Fig. 4:**
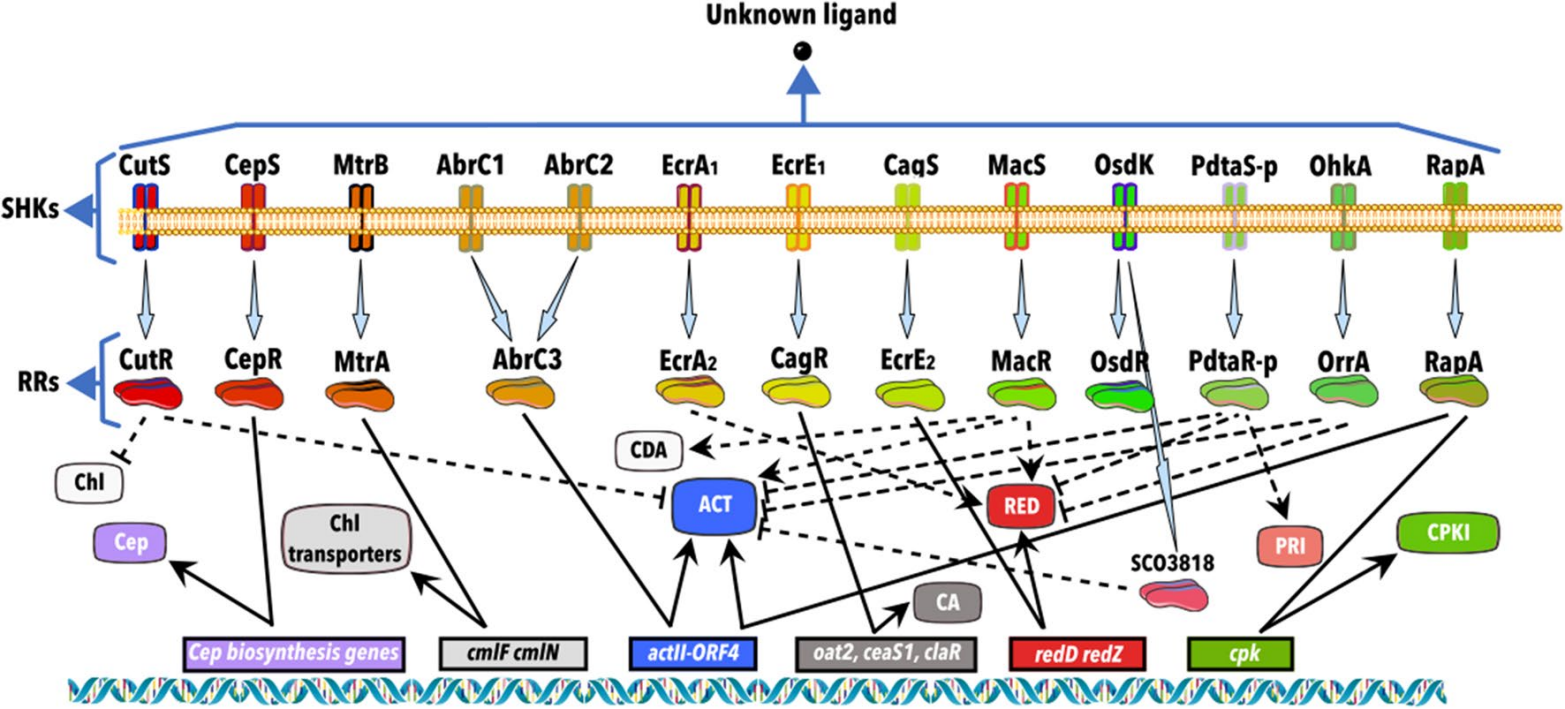
A schematic representation of the regulation pathways by the two-component systems with an unknown trigger signal is illustrated. An arrow connects Sensor Histidine Kinases and their cognate response regulators. Lines that end with an arrow indicate a positive regulation, and truncated lines indicate a negative regulation. Straight lines indicate a direct effect through genes involved in synthesizing or regulating the metabolite, and dotted lines indicate indirect or unknown regulation. ACT, actinorhodin; CA, clavulanic acid; CDA, calcium-dependent antibiotic; Cep, cephamycin; Chl, chloramphenicol; CPKI, coelimycin polyketide synthase I; PRI, pristinamycin; and RED, undecylprodigiosin

The first atypical TCS with two different SHKs in *Streptomyces* was described in *S*. *coelicolor.* In this example, the genes that code for AbrC1/C2/C3 (*sco4598*, *sco4597*, and *sco4596*) (Fig. 4) are separated by DNA regions long enough to harbor their promoters (114 bp and 308 bp, respectively). Thus, each gene could be expressed independently, responding to the different bacteria’s needs. Deletion of the entire cluster impaired the production of ACT, RED, and CDA, with normal morphological development. The results were similar in a single *abrC3* mutant, but the CDA production was unaffected. This last discovery suggested that the additional SHK might specifically phosphorylate the RR involved in CDA production. The targets of AbrC3, demonstrated by the combination of ChIP-chip and microarray experiments, were *actII*-*ORF4* and the genes *afsS* and *absR1*, also involved in antibiotic regulation (Rico et al. 2014a). To obtain more information about the individual role of each SHK and the RR, RT-qPCR and autophosphorylation experiments were performed utilizing single mutants. However, these experiments did not give a differential expression of *abrC1* and *abrC2*, supporting that there is a single promoter for both genes. Even so, AbrC3 autoregulates this promoter. Therefore, co-transcription of both SHKs suggests (i) that these are important in sensing external stimuli, (ii) that their constitutive expression is different along time in a media-dependent manner, and (iii) that its regulation likely occurs at the post-translational level (Rodríguez et al. 2015).

MtrAB (*sco3013*-*sco3012*) (Fig. 4) is a TCS highly conserved in *Actinobacteria*, likely involved in coordinating chloramphenicol production and sporulation in *Streptomyces* *venezuelae* NRRL B-65442. Using ChIP-seq, it was demonstrated that MtrA binds to DNA between the divergent *cmlN* and *cmlF* regions, which encode for transporters required for chloramphenicol production. It also binds to the divergent *jadR1* and *jadR2* genes, which repress jadomycin biosynthesis. Also, they cross-regulate chloramphenicol production. Other genes regulated by MtrA are *bldN*, *whiBDEGH*, and *ssgABDEG*, involved in sporulation and differentiation, respectively. In *ΔmtrB* mutants, MtrA is activated, causing a global shift in the metabolome, including a constitutive and high chloramphenicol production (Som et al. 2017b). Furthermore, ChIP-seq experiments demonstrated that *S*. *coelicolor* also regulates developmental genes and the genes that encode ActII-1, ActII-4, and RedZ, which are the regulators of ACT and RED makes this TCS a key regulator of antibiotic production (Som et al. 2017a). Also, it has been demonstrated that the phosphorylation site D53 in the RR is crucial for binding the regulated genes (Lu et al. 2022).

In *Streptomyces* *clavuligerus* F613-1, CepRS (*BB341*_*RS13780*/*13785*) (Fig. 4) is a TCS involved in cephamycin C production. Single and double mutant strains were studied by RT-qPCR and EMSA experiments, supporting that the absence of CepRS reduces cephamycin C production by regulating the genes involved in the early, middle, and late stages of its biosynthesis (*pcbC*, *pcbAB*, *lat*, *cefD*, *cefE*, *cmcI*, *cmcJ*, *cefF*, and *cmcH*). CepR interacts with the *cefD*-*cmcI* intergenic region as a transcriptional activator of cephamycin C but not in the clavulanic acid (CA) biosynthesis, which is simultaneously produced during the *S*. *clavuligerus* F613-1 fermentation (Fu et al. 2019a). The pathway of CA formation is well studied.

The TCS CagRS (Fig. 4), coded in *orf22* and *orf23* of *S*. *clavuligerus* F613-1 (located next to the CA biosynthetic gene), and the clavam gene clusters appear to be involved in their global regulatory mechanism (López-Agudelo et al. 2021). Deletion of CagRS resulted in decreased production of CA, and transcriptome. ChIP-seq experiments also supported that the genes involved in the primary metabolism could be regulated by CagR, including those for glyceraldehyde 3-phosphate (G3P) metabolism and arginine biosynthesis, which interestingly are precursors of CA. This result was probed by EMSA experiments, observing that CagR directly binds to genes such as *arG*, *oat1*, *oat2*, *ceaS1*, and *claR*, indicating that this TCS is a pleiotropic regulator that directly affects CA biosynthesis and indirectly regulates the metabolism of arginine and G3P. This study shows that CagR binds to the promoter region of *BB341_rS25520* (*avaA2*) gene, which encodes for a gamma-butyrolactone biosynthesis protein (Fu et al. 2019a,b). This signaling system negatively regulates CA production by inhibiting *ccaR* (Santamarta et al. 2005), suggesting that butyrolactone may be the signal molecule for this TCS (Fu et al. 2019b). Still, additional studies are needed to confirm this.

ChIPseq, footprinting, and EMSA assays demonstrated that MacRS (*sco2120* and *sco2121*) from *S*. *coelicolor* (Fig. 4) positively and indirectly regulates the ACT, RED, and CDA biosynthesis, respectively. Liu et al. (2019) identified a consensus sequence for regulating genes involved in morphogenesis. Also, functional conservation for this TCS was observed by successfully using *S. avermitilis* and *S. venezuelae* homologs as substitutes for MacRS (Liu et al. 2021). On the other hand, the biosynthesis of the antifungal compound natamycin produced by various *Streptomyces* species, including *Streptomyces gilvosporeus*, appears to be controlled positively by MacRS (*rs12540* and *rs12545*). Thus, qPCR experiments in *∆macRS* mutants showed that the expression of *sgnS2*, *sgnS0*, *sgnA*, *sgnD*, *sgnT*, and *sgnK* was significantly reduced, and the production of natamycin was undetectable (Zong et al. 2022).

Autophosphorylation and transphosphorylation assays with ^32^P-radiolabeled ATP allowed the description of OsdR, an *S. coelicolor* RR homolog of the DevR RR from *Mycobacterium tuberculosis.* OsdR (*sco0204*) could be activated by the SHK OsdK (*sco0203*) (Fig. 4). In silico analysis and in vitro DNA binding assays revealed that this RR binds to the upstream stress-related genes (*sco0167-sco0219*) associated with the stress control and development of *S. coelicolor* (Urem et al. 2016). Moreover, it is known that OsdK has a second cognate RR coded by *sco3818*, and the deletion of any of these enhances the production of ACT (Wang et al. 2009).

Initially, the orphan SHK PdtaS-p (SSDG_02492) of *Streptomyces pristinaespiralis* (Fig. 4) was identified to positively regulate the production of pristinamycin (PI and PII) and negatively ACT and RED (Wang et al. 2015; Li et al. 2020). Later by bioinformatic analyses, the use of Δ*pdtaR-p* and Δ*pdtaS-p* mutants and phospho-transfer assays, the orphan RR PdtaR-p (SSDG_02492) was identified as its cognate RR. Interestingly, this RR has the particularity of containing a putative ANTAR RNA-binding domain that works as an antiterminator at the post-transcriptional level (Li et al. 2020).

OhkA (*sco1596*) is also an orphan SHK of *S. coelicolor.* Its cognate RR OrrA (*sco3008*) (Fig. 4) was identified in mutants where the absence of any of the two components led to increased production of ACT and RED. Its transcriptomic analysis revealed that genes involved in the biosynthesis of both antibiotics were affected, among others. The interaction between these two proteins was determined by two-hybrid system assays (Zheng et al. 2021).

Deletion of RapA1/A2 (sco5403/*sco*5404) TCS in *S. coelicolor* (Fig. 4) reduced ACT production. A proteomic study revealed a decrease in a CpkI protein. RT-PCR assays showed that this regulation occurs through the genes *actII-orf4* and *kasO*. However, it is still necessary to characterize the *cpk* cluster product, to correlate the gene transcript abundance with changes in coelimycin production (Lu et al. 2007; Bednarz et al. 2019).

In *S. coelicolor*, the genes *ecrA1* and *ecrA2* (*sco2518* and *sco2517*), and *sco6421* and *sco6422* code for two TCS involved in the positive regulation of RED biosynthesis (Yong-Quan et al. 2004). A transcription analysis with northern blotting indicated that this regulation occurs through *redD* and *redZ* (Wang et al. 2007).

The proteins of the CutRS TCS in *S. lividans* were analyzed with bioinformatic protein tools, and the similarity with the OmpR and EnvZ proteins from *E. coli* was identified (Tseng and Chen 1991). The negative regulation over ACT production (Du et al. 1996) and chloramphenicol (McLean et al. 2019b) has been reported.

The orphan RR Aor1 (sco2281) in *S. coelicolor* is co-transcribed in an operon which includes two hypothetical proteins and a TetR transcriptional regulator (sco2279, sco2282, and sco2280, respectively). By deletion of Aor1 and RNA-seq experiments, it has been suggested that this RR is a positive regulator of ACT, RED, and CDA production. Still, the direct target genes have yet to be elucidated, and although various SHKs are partner candidates for this RR, it has been a difficult task to identify them (Antoraz et al. 2017).

## Challenges in ligand identification

**Fig. 5:**
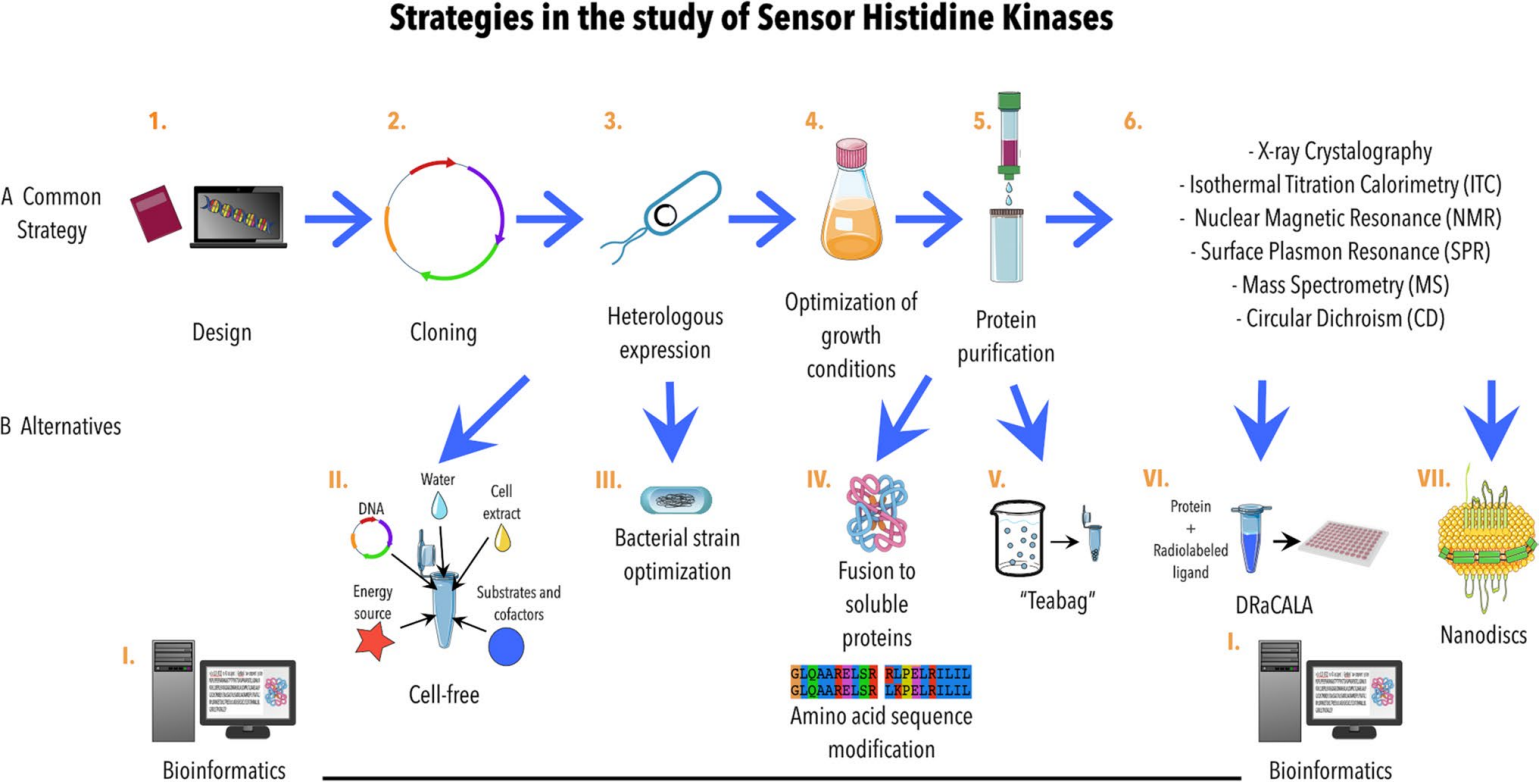
(**A**) The most common strategies when studying Sensor Histidine Kinases: 1. Experimental design. 2. Cloning of the coding sequence into the adequate vector. 3. Transformation in the selected bacterial strain for heterologous expression. 4. Optimization of growth conditions and detection of the protein expression (this includes culture media, temperature, incubation time, inducer concentration, etc.). 5. Protein purification (selection of detergents for solubilization and methods for purification like dialysis, ultracentrifugation, chromatography, etc.). 6. Submission of the protein to different methodologies for detection of protein–protein, protein–ligand interactions and/or structure determination. (**B**) Some of the alternatives presented in this review are illustrated: I. Bioinformatics is useful along the process, from experimental design modifying coding sequences or adding fusion proteins, to predictions of the structure and ligand or protein binding. II. Cell-free could let avoid the issues when expressing heterologously. III. Bacterial strain optimization uses the principle of a repressor before the addition of the inductor for the T7 RNA polymerase for example. IV. Modifications on the amino acid sequence and the fusion to soluble proteins are used to solubilize the protein and facilitate the purification process. V. Emerging technics like “teabag” method seeks to optimize the purification process utilizing affinity resins. VI. DRaCALA has emerged as an alternative for the protein–ligand detection with some advantages but using radioactivity. VII. Nanodiscs also present some technical difficulties but the promise to maintain the membrane proteins in their native conformation and the use of polymers can make it interesting in the future

The SHKs can detect a remarkable diversity of chemical and physical signals, like small ligand nutrients, temperature changes, or even mechanical forces (Dufrêne and Persat 2020). SHKs are localized in the extra cytoplasmatic space, the membrane, and the cytoplasm (Zschiedrich et al. 2016). Due to their high hydrophobicity, these proteins are slightly soluble in aqueous environments (Rawlings 2016), complicating the most common strategies to express and study them. Methodologies to characterize SHKs rely on obtaining the heterologous protein in *E. coli* as a host (Fig. 5A). However, when the expression is achieved, the yield can still be (around 0.1–3 mg/L) compared to other soluble proteins condition that limits their characterization. To increase protein production is necessary to optimize growth conditions (medium, temperature, incubation time, inducer, etc.), solubilization (detergent selection) as well as purification strategies (Ma and Phillips-Jones 2021) (Fig. 5B). Furthermore, these proteins sometimes are also misfolded forming aggregates and inclusion bodies. Besides, proteins that bind to the membrane need to be extracted by detergents (Rawlings 2016). All these are the classic technical challenges faced when working with hydrophobic membrane proteins. Still, for SHKs, a further concern is the requirement of high yields of the intact protein (and properly folded) necessary for in vitro screening to identify ligands and inhibitors.

Nanodisc membranes and proteoliposomes have been successfully used to investigate interaction parameters between SHKs, their cognate response regulator, and their accessory protein (Hörnschemeyer et al. 2016). Despite the advantages offered by this technology, some limitations are present since nanodiscs reconstitution and extraction of membrane proteins requires detergents, affecting the lipid bilayer composition and, therefore, the protein structure, activity, and regulation (Chen et al. 2020).

Surface plasmon resonance (SPR), isothermal titration calorimetry (ITC), mass spectrometry (MS), and circular dichroism (CD) are techniques typically used for detecting protein–ligand interactions (Hussain et al. 2016). For example, ITC requires high amounts of protein, allowing the detection of low molecular weight molecules binding to the protein. It is also necessary to consider protonation-deprotonation reactions and various conformational states which can affect the reactivity (Paketurytė et al. 2019). Furthermore, these methods require specialized equipment and experienced personnel capable of interpreting the results (Williams and Daviter 2013).

Nuclear magnetic resonance (NMR) and X-ray crystallography are constantly updated and applicable to study protein–ligand interactions. Still, the inherent technical difficulties, such as the low quality of crystals due to protein-detergent complexes, hamper the characterization of these proteins (Maslennikov et al. 2010). In recent years, crystallized structures of several SHKs have been obtained. This information, bioinformatic studies, and protein engineering allowed elucidation of their interaction with RRs. Nevertheless, due to their complex domain architecture, many questions remain about how the ligand is detected and transmitted through the membrane into the cytoplasm (Jacob-Dubuisson et al. 2018). These and, in some cases, the need for specialized equipment and the economic limitations can be problematic bottlenecks to overcome in laboratories worldwide (Fig. 5B).

## The use of bioinformatic tools

Recent advances in bioinformatics combined with structural studies have facilitated our functional and mechanistic understanding of TCSs. Dynamic simulation studies have allowed the prediction of structures, conserved functional domains, and likely target genes (Zschiedrich et al. 2016). An example is the identification and classification of the TCSs in *S. venezuelae* NRRL B-65442 by the P2RP program. This user-friendly online tool identifies regulatory proteins, including TCSs, by reverse PSI-BLAST. It searches the query sequences against SMART and Pfam databases (McLean et al. 2019a) and computes the secondary structure of regulatory proteins using PSIPRED (Barakat et al. 2013). This tool predicted that *S. venezuelae* NRRL B-65442 has 58 TCSs, 26 unpaired SHKs, and 17 RRs.

The pangenome of 93 sequences of *Streptomyces* species has been analyzed using a Bacterial Pan Genome Analysis tool (BPGA) (Chaudhari et al. 2016). This tool identified 15 TCSs highly conserved, 12 of which have a predicted or known function by homology with the TCSs in other genera (McLean et al. 2019a). Another example is a tree constructed using the maximum likelihood method and the Le Gascuel model. This method allows inferring the relation among RRs, applying the neighbor-join and BioNJ algorithms in *MEGA X*.

A model of AbrB1 RR was performed in *SWISS-MODEL* using the VraR structure as a template. This method permitted the classification of AbrB1 as part of the NarL family, characterized by a REC receiver domain and an HTH-LuxR DNA-binding domain. On the other hand, AbrB2 showed a putative classic architecture with three transmembrane regions, an extracellular input domain, a HisKa-3 domain, and a HATPase-c domain. Furthermore, it indicates that AbrB1/AbrB2 is highly conserved in *Streptomyces* spp. with a > 75% identity in the case of RR (Sánchez de la Nieta et al. 2020).

Bioinformatics tools have been used successfully in other microorganisms for TCSs studies. An example is *Myxococcus xanthus*, a Gram-negative deltaproteobacterium forming spores in response to starvation. Using bioinformatics in combination with functional analyses evidenced the unusual organization of the TCSs, where 71% corresponded to orphan genes. Bioinformatics suggests that the interaction of these proteins and the complex gene clusters makes the functionality different from encoded pairs. Experimental evidence evidenced that 25 orphan SHK were transcriptionally upregulated during development. Two are essential for viability, and four new SHKs are important in spore germination (Shi et al. 2008). Several examples of knowledge gained through combined structural and bioinformatics studies are cited in Zschiedrich et al. (2016), making it evident that the progress of these bioinformatics tools facilitates improvement in the study of TCSs. Also, many efforts have been devoted to making devices capable of predicting the TCSs encoded in the bacterial genomes (Balewski and Hallberg 2019) like pathogens causing life-threatening nosocomial infections (Rajput et al. 2021) and plants like soybean (Mochida et al. 2010) and chickpea (Ahmad et al. 2020).

Furthermore, molecular docking methods can predict the match for a ligand and a protein generating several conformations/orientations of the ligand within the protein. One advantage is that the three-dimensional structure can be deduced from an experimentally solved structure or obtained by homology modeling (Salmaso and Moro 2018). Furthermore, molecular docking methods can predict the match for a ligand and a protein generating several conformations/orientations of the ligand within the protein. An example is the pathogenic Gram-positive bacterium *Staphylococcus aureus.* The application of molecular modeling, molecular dynamics simulation, and MM/GBSA determined that the Walk of *S. aureus* SHK has a potent inhibitor known as waldiomycin. It was also possible to postulate that Lys100 is crucial for the great affinity for this compound (Radwan and Mahrous 2020).

These examples make to consider that the progress in bioinformatic tools is promising in studying TCS on *Streptomyces* and other microorganisms.

## Alternatives in the study of SHKs

While studying the TCSs, a common problem that needs to be surpassed is the characterization of the SHK and the identification of the ligand that triggers its response. This could be achieved by generating tools that efficiently produce protein for the needed experiments. An interesting approach for the heterologous expression of membrane proteins is the addition of soluble fusion proteins and the computational protein redesign to make them soluble in water facilitating its purification and bringing good stability to subsequent studies. However, using these fusion proteins can make crystallization procedures difficult, and modifying the amino acid sequence can affect the protein’s functionality (Rawlings 2016). Regarding the *E. coli* heterologous system expression, BL21(DE3) is the most employed strain in combination with the T7 RNA polymerase (RNAP). Still, when working with some toxic membrane proteins, a different approach is needed, such as the Lemo21(DE3) strain in which the membrane proteins toxicity is minimized (Schlegel et al. 2012). In this regard, overexpression of two multidrug ABC transporters (BmrA, homodimer from *Bacillus subtilis*, and PatA/PatB, heterodimer from *Streptococcus pneumoniae*) was compared in different *Escherichia coli* strains. The results suggested that the structure and activity of these proteins are influenced by the amount of T7 RNAP, allowing high amounts of biomass, cell survival, and good levels of heterologous protein expression (Mathieu et al. 2019). In this system, the leakage expression of the T7 RNAP was under the control of inducible promoters like PrhaBAD and Ptet. This situation prevents the rapid production of recombinant proteins from the inducing period and allows the cell to use nutritional resources for its growth (Du et al. 2021).

Another option to overcome the problem of protein yields obtained in the heterologous expression of SHKs is the optimization of techniques such as *cell-free* to produce membrane proteins. In this methodology, we eliminated the need for cell viability (Khambhati et al. 2019). This system enhances the production of proteins, using only cell raw materials. These materials include RNA polymerase, ribosomes, tRNA synthases, translation factors, nucleotides, amino acids, energy sources (phosphoenolpyruvate, glucose-6-phosphate, or fructose 1,6-bisphosphate, among others), and plasmid DNA as a template, containing the coding sequence for the interested protein (Khambhati et al. 2019). In the study of membrane proteins from *E. coli*, this methodology, in combination with the use of inner membrane vesicles, has been successfully used to express two complex integral membrane proteins (tetracycline pump and mannitol permease) with yields up to 400 times in comparison with the in vivo system (Wuu and Swartz 2008). Efforts have been made to optimize this system for genes with a high GC content. For instance, in *S. lividans*, the concentration of the EGFP protein has reached yields up to 116.9 ± 8.2 µg/ml (Li et al. 2017a, 2018). In *S. venezuelae*, an optimized procedure for synthesizing various proteins (like OTC and NRPSs) was established, obtaining a reasonable number of metabolites that were easy to purify and analyze (Moore et al. 2021). Although the abovementioned methodology has been commonly used in the biosynthesis of natural products such as NRPs and RiPPS (Ji et al. 2022), it is worth exploring this technique for synthesizing SHKs from *Streptomyces*.

Another novel method, named “teabag,” promises to reduce the purification times to obtain high-quality proteins by utilizing affinity resins within a porous container that does not need expensive equipment. With this method, purifying five different membrane proteins from bacteria, humans, and marsupials, which have been expressed heterologously in *E. coli*, *Saccharomyces cerevisiae*, and insect cells, has been possible. These purified proteins had the necessary quality for further applications like ITC, crystallization, and cryogenic electron microscopy (Hering et al. 2020).

Some other approaches are being focused on eliminating the need for purified protein, such as the widely used differential radial capillary action of ligand assay (DRaCALA). In this assay, lysates from expression library cells overexpressing the interested proteins can be rapidly analyzed in 96-well plates for protein–ligand interactions (Roelofs et al. 2011; Orr and Lee 2017). This methodology has proved that the SHK from the TCS YehU/YehT (renamed BtsS/BtsR) from *E. coli* is a high-affinity receptor for extracellular pyruvate (Behr et al. 2017). However, some of the disadvantages of this procedure are radioactive labeling, which requires specific conditions for its handling and cannot be applied to all molecules. Regarding this problem, recent alternatives that eliminate this requirement promise to make it more accessible (Cimdins-Ahne et al. 2022).

In conclusion, the genus *Streptomyces* contains some of the largest bacterial genomes. The highest number of TCSs described is encoded by these bacteria, reflecting the need for adaptation to their highly variable environment (McLean et al. 2019a). In this process, the production of secondary metabolites is essential for their proper growth, and their biosynthesis is mediated by complex regulatory cascades that regulate pathway-specific switches (Romero-Rodríguez et al. 2018). TCSs are indispensable in linking environmental stimulus to a transcriptional response (Romero-Rodríguez et al. 2015). Until 2019 of the predicted 58 TCSs of *S. venezuelae*, only 22 have been partially characterized (McLean et al. 2019a). This condition may be because the study of signal transduction between the SHKs and RRs domains represents a major challenge because of the size, flexibility, and dynamics of SHKs (Jacob-Dubuisson et al. 2018). Implementing techniques such as RT-qPCR, ChIP-seq, CRISPR-Cas9, and bioinformatics have allowed further progress in understanding the regulatory response. But even so, there is still much work to do, not only to describe the RRs but also to identify the signals that generate their response through the SHKs. The answers to these questions will unveil the TCSs role within the complex regulatory network in* Streptomyces.*

The approaches of controlling the expression of proteins in heterologous models such as *E*. *coli* and using recent methods like the *cell-free* system will allow the production of enough protein quantities to study and characterize them. But there is still the problem of the accessibility to methods that enable structure determination and ligand–protein interactions. The development of technologies like nanodiscs which resulted in the use of polymers that could finally eliminate the use of detergents and maybe extend their biotechnology applications (Chen et al. 2020) is a clear example of the continuous search for alternatives in the study of transmembrane proteins. A combination of bioinformatic tools and the constant development of new experimental techniques is essential for continuing to generate knowledge and understanding of TCSs. These results will enhance the production of industrially secondary metabolites and could facilitate the discovery of novel compounds.

## Supplementary Information

Below is the link to the electronic supplementary material.

Supplementary file1 (PDF 218 KB)
