# Supplementary material for: Trends in the two-component system’s role in the synthesis of antibiotics by Streptomyces
Source: Appl Microbiol Biotechnol. 2023 Jun 21;107(15):4727–43. doi: 10.1007/s00253-023-12623-z (PMC10345050; doi:10.1007/s00253-023-12623-z)
Supplement: Supplementary file 1 — Supplementary file1 (PDF 218 KB) [file 253_2023_12623_MOESM1_ESM.pdf]

Supplementary Material for

Applied Microbiology and Biotechnology

Trends of the two-component system's role in the synthesis of antibiotics by *Streptomyces*

Rodrigo Cruz-Bautista*, Beatriz Ruíz-Villafán, Alba Romero-Rodríguez, Romina Rodríguez-Sanoja, and Sergio Sánchez*.

Instituto de Investigaciones Biomédicas, Universidad Nacional Autónoma de México. Ciudad Universitaria, CdMx, 04510. México.

*Corresponding authors. Rodrigo Cruz-Bautista, rodyac@outlook.com
Sergio Sánchez, sersan@biomedicas.unam.mx

Table S1. Antibiotics stimuli and regulation exerted by TCS in *Streptomyces* species.

Name	*Streptomyces* species	Stimuli in medium	Regulation	Reference
PhoP/R	*S. lividans*<br><br>*S. roseosporus*<br><br>*S. hygroscopicus* var. *geldanus*<br><br>*S. tsukubaensis*	Phosphate	↓ ACT and RED (via AfsR and RpoZ).<br><br>↓ Daptomycin (via AtrA and AdpA)<br><br>↓ Geldanamycin	Matín et al. 2017.<br><br>Zheng et al. 2019.<br><br>Martín et al. 2019.
ChiR/ChiS	*S. thermoviolaceus*<br><br>*S. lividans*	Chitobiose and chitin	↑ Chi40 (chitinase)	Homerová et al. 2002.
SCO5784/SCO5785	*S. coelicolor*	Glucose	↑ ACT, RED*	Rozas et al. 2012.
SCO3134*	*S. coelicolor*	Glucose	Unknown	Romero-Rodríguez et al. 2016a
SCO4020/SCO4021	*S. coelicolor*	Glucose	Unknown	Romero-Rodríguez et al. 2016a
SCO6162/SCO6163	*S. coelicolor*	Glucose	↓ ACT, RED	Cruz-Bautista et al. (in preparation)
Dra-R/K	*S. coelicolor*<br><br>*S. avermitilis*	Aminoacids	↑ ACT (*actII-ORF4*), ↓RED and yCPK (*kasO*)<br><br>↓ Avermectin<br>↑ Oligomycin (*olmRI*)	Yu et al. 2012.
AfsQ1-Q2<br><br>RspA1A2<br><br>AfrQ1Q2	*S. coelicolor*<br><br>*S. albus*<br><br>*S. rimosus*	Glutamate<br><br>Soybean oil<br><br>Glycine	↑ ACT, RED, CDA (*actII-ORF4*, *redD* and *cdaR*) and coelimycin P2<br><br>↑ Salinomycin (*slnR*)<br><br>↓ Oxytetracycline (*oxy*)	Shu et al. 2009 and Chen et al. 2016.<br><br>Zhang et al. 2021.<br><br>Ni et al. 2020.

GluR-GluK	*S. coelicolor*	Glutamate	↑ RED, yCPK and ↓ ACT	Li et al. 2017.
RapA1/A2<br><br>RimA1/A2	*S. coelicolor*<br><br>*S. rimosus*	Unknown<br><br>Glycine	↑ ACT (*actII-ORF4*) and a type I polyketide (*kasO*)<br><br>↓ Oxytetracycline (*oxy*)	Bednarz et al. 2019.<br><br>Ni et al. 2019.
DevS/R	*S. coelicolor*	Nitric oxide	ACT (*actII-ORF4*)	Honma et al. 2021
AbrA1/A2	*S. coelicolor*	$MgSO_4$ and $FeSO_4$	↓ ACT, RED and CDA.	Rico et al. 2014b.
VanR-VanS<br><br>AbrB1/B2	*S. coelicolor*<br>*S. toyocaensis*<br><br>*S. coelicolor*	Vancomycin	↑ vancomycin resistance (*vanSRJKHAX*)<br><br>↓ ACT (*scbR2*), RED (*red*), ↑ vancomycin resistance (*van*) and coelimycin (*rapA1*/*rapA2*)	Hutchings et al. 2006 and Novotna et al. 2016.<br><br>Sánchez de la Nieta et al. 2020.
AbrC1/C2/C3	*S. coelicolor*	Unknown	↑ ACT and RED (*actII-ORF4, afsS*)<br><br>↑ CDA (via AbrC3 and an unknown RR)	Rico et al. 2014a.
MtrAB	*S. coelicolor*<br><br>*S. venezuelae*	Unknown	ACT and RED (*actII-ORF1*, *actII-ORF4* and *redZ*)<br><br>↓ Chloramphenicol (*cmlN* and *cmlF*)<br><br>Jadomycin (*jadR1* and *jadR2*)	Som et al. 2017a.<br><br>Som et al. 2017b.
CepRS	*S. clavuligerus*	Unknown	↑ Cephamycin (*cefD-cmcI*)	Fu et al. 2019b.

MacRS	*S. coelicolor*<br><br>*S. gilvosporeus*	Unknown	↑ ACT, RED and CDA<br><br>↑ Natamycin (*sgnR*)	Liu et al. 2019.<br><br>Zong et al. 2022.
OsdK-OsdR-SCO3818	*S. coelicolor*	Unknown	↑ ACT	Wang et al. 2009 and Urem et al. 2016.
PdtaS-p - PdtaR-p	*S. pristinaespiralis*	Unknown	↑ Pristinamycin<br><br>↓ ACT and RED	Li et al. 2020.
OhkA-OrrA	*S. coelicolor*	Unknown	↓ ACT and RED	Zheng et al. 2021.
EcrA1/EcrA2	*S. coelicolor*	Unknown	↑ RED (*redD*, *redZ*)	Wang et al. 2007.
CutRS	*S. lividans*	Unknown	↓ ACT and Chloramphenicol	Chang et al. 1996 and McLean et al. 2019b.
Aor1*	*S. coelicolor*	Unknown	↑ ACT, RED and CDA	Antoraz et al. 2017.

- Arrows indicate ↑ positive and ↓ negative regulation. If not stated, the regulation has not yet been established. TCSs in the same box are considered homologs. Names of the genes and proteins between parentheses indicate the pathway regulation. If not stated, the regulation is indirect or unknown. *Orphan response regulators.
